# On the outcomes of teacher wellbeing: a systematic review of research

**DOI:** 10.3389/fpsyg.2023.1205179

**Published:** 2023-07-27

**Authors:** Benjamin Dreer

**Affiliations:** Erfurt School of Education, University of Erfurt, Erfurt, Germany

**Keywords:** teacher wellbeing, systematic review, effects, teacher retention, teacher-student relationships, student outcomes

## Abstract

**Introduction:**

Teacher wellbeing is a growing area of research that has seen a steady increase in publications in recent years. The subsequent need to synthesize and structure this existing research has been articulated and addressed by a handful of systematic research reviews. However, no previous reviews have examined the potential outcomes of teacher wellbeing as a primary theme.

**Methods:**

Following the preferred reporting items for systematic reviews and meta-analyses (PRISMA) guidelines, this review has identified and evaluated the studies investigating the possible outcomes of teacher wellbeing. A keyword search identified 397 records. After the records were screened, 44 research studies analyzing data from over 76,990 teachers were included in this in-depth analysis; the concepts, methods and findings of these studies were examined.

**Results and discussion:**

The results of this review highlight the significant relationship of teacher wellbeing with several factors and desirable outcomes, including teachers' sleep quality, teacher retention, teacher–student relationships, and student outcomes. However, only a few of the included studies employed methodologies that support causal interpretations of these effects. In light of the present findings, this paper offers three main recommendations to support future progress in this field.

## 1. Introduction

The last 10 years have seen a large increase in the number of publications exploring teacher wellbeing (McCallum et al., 2017; Hascher and Waber, 2021). Interest in this topic has grown for a reason. Reports of high attrition rates and teacher shortages from all over the globe (Cano et al., 2017) have prompted educational researchers to seek knowledge and solutions that can improve job satisfaction, reduce burnout, and increase retention among teachers. In the literature, there is little consensus on how wellbeing should be defined. One commonly accepted view is that wellbeing is a multidimensional concept. Diener's (1984) seminal work introduced a two-component model of wellbeing, incorporating cognitive factors such as life satisfaction and affective elements like positive and negative affect. Subsequently, various multi-component models such as those proposed by Ryff (1989); Warr (1999); Keyes (2002), and Seligman (2011) have emerged. Other approaches, such as the job demands-resources model (Demerouti et al., 2001) and the ecological framework (McCallum, 2020) aim to advance the understanding of wellbeing by considering the specific contexts of the workplace. After reviewing the pertaining concepts, Viac and Fraser (2020) offered a basic definition of teacher wellbeing, that is, “teachers' responses to the cognitive, emotional, health and social conditions pertaining to their work and their profession” (p. 18). To maintain a broad perspective and avoid prematurely focusing on one specific theoretical framework, this umbrella definition has been embraced as an initial starting point.

In recent years, there has been a growing focus on investigating factors linked to teacher wellbeing. However, this emphasis is predominantly seen from a theoretical standpoint that prioritizes contributions to wellbeing. Despite the prevalent use of correlational analyses in these studies, there has been comparatively less emphasis on exploring associations as potential outcomes of wellbeing. Research on employees in other fields has identified the positive impacts of wellbeing that reach far beyond the individual psychological benefits and employee retention, as wellbeing is also associated with increased productivity and innovation across entire enterprises (Carolan et al., 2017; Katebi et al., 2022). However, the spectrum of the effects of teacher wellbeing remains largely unknown. Though early researchers in this area seem to have recognized that teacher wellbeing could impact teaching behavior and workplace relationships (McCallum and Price, 2010), a more comprehensive scope of the potential effects of wellbeing has not yet been systematically explored.

**Figure 1:**
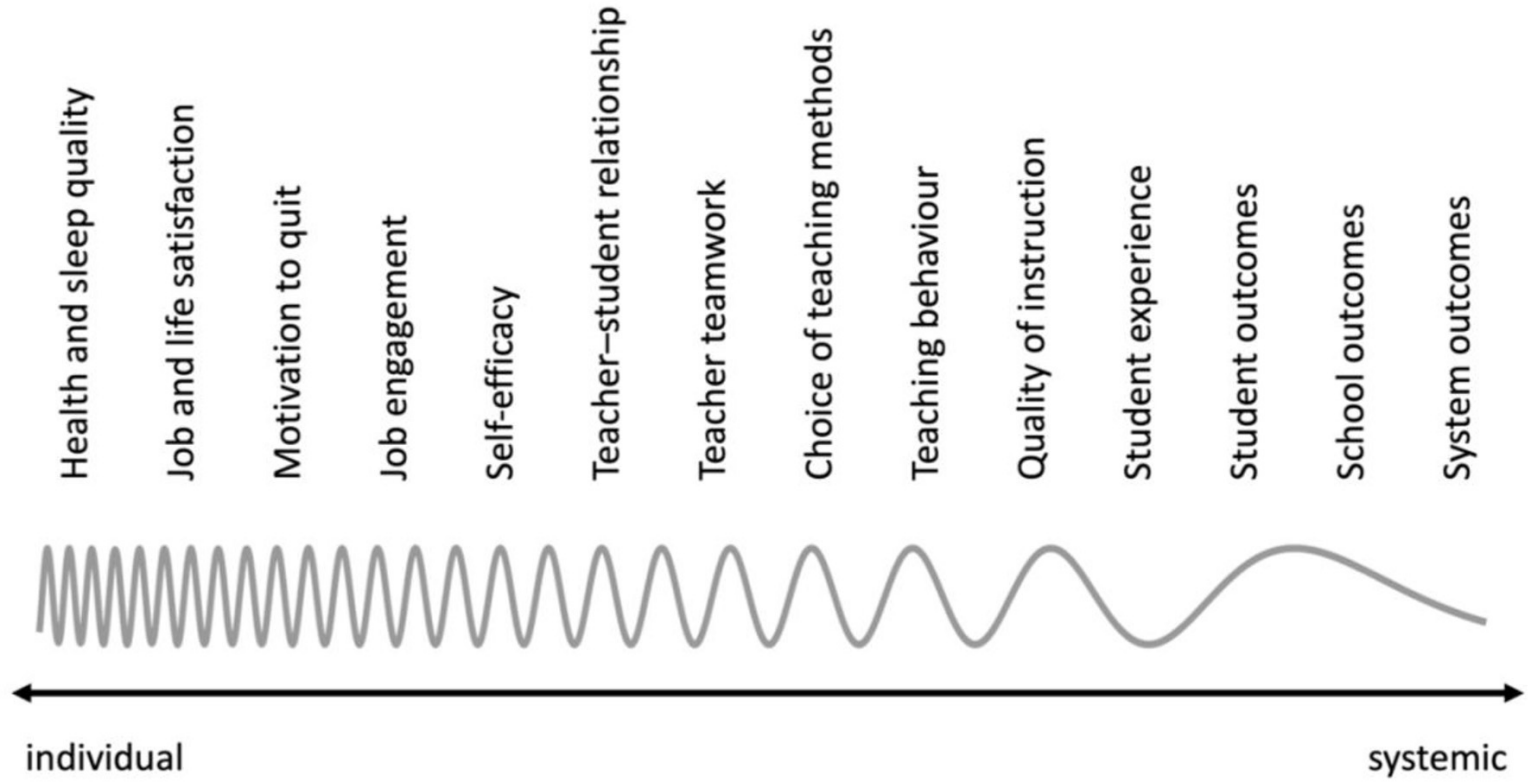
Spectrum of the potential outcomes of teacher wellbeing.

Existing theoretical frameworks suggest that teacher wellbeing could have a broad spectrum of potential effects (see Figure 1). For example, the theoretical framework of job demands and resources (Demerouti et al., 2001) suggests a link between teacher wellbeing and teacher satisfaction. Generally, this theory posits that teachers can better and more successfully meet the demands of their jobs if they are able to take advantage of the appropriate resources. Job resources may buffer the impact of the demands at work on burnout (Bakker et al., 2005). More specifically, Skaalvik and Skaalvik (2018) hypothesized that teacher wellbeing, which may be considered a type of job resource, predicts higher job engagement and satisfaction and lower motivation to leave the profession.

Based on the broaden-and-build theory (Fredrickson, 2001), Frenzel (2014) assumed that teachers who more frequently experience positive events during everyday school life are more able to build good working relationships, are more open to new experiences and methods, and deal with uncertainties and obstacles more flexibly. Conversely, teachers who experience more negative emotions in the workplace will be less likely to actively engage in relationships, will stick to familiar methods and routines and will struggle to deal with unforeseen obstacles. This theoretical approach highlights the ways that the characteristics of teacher wellbeing can be linked to teacher behavior inside and outside the classroom.

A mechanism called emotional contagion further supports these assumptions. As defined by Hatfield et al. (1993), emotional contagion describes “the tendency to automatically mimic and synchronize expressions, vocalizations, postures, and movements with those of another person's and, consequently, to converge emotionally” (p. 96). Several studies have supported the view that this takes place in classrooms, as well (Becker et al., 2014; Houser and Waldbuesser, 2017). For example, when teachers smile at their students, the students are—due to the behavioral reflex of mimicry—likely to smile back. Because of this physical reaction, students are then apt to feel increased happiness (feedback), which, in turn, can positively impact motivation and learning. Mottet and Beebe (2000) hypothesized that emotional contagion has a greater impact on non-verbal classroom behavior than on verbal behavior.

The model of the prosocial classroom (Jennings and Greenberg, 2009) posits that teacher wellbeing is one primary factor influencing healthy teacher–student relationships, effective classroom management, and effective social-emotional learning, all of which are directly linked to a healthy classroom climate and to students' social, emotional, and academic outcomes. This model also proposes that these factors directly or indirectly influence teacher wellbeing, indicating a relation of interdependence among teachers, students, and classroom outcomes.

Although a broad spectrum of the potential impacts of teacher wellbeing can be deduced from established theories, no systematic overview of studies investigating these potential effects has yet been conducted, as previous reviews of research in this field have focused on other issues. It is expected that more evidence will be available for outcomes that are closer to the individual, as such effects of teacher wellbeing might be easier to detect. The more systemic the outcomes, the more difficult it might be to uncover potential effects, as they may be distorted on their pathway through sub-systems. This is illustrated by the wavy line in Figure 1.

## 2. Previous reviews

Some reviews that gathered and structured previous research on the topic of teacher wellbeing have been published in recent years. Bricheno et al. (2009) reviewed the nature of teacher wellbeing, including the concepts, arguments, contributing factors, and interventions targeting teacher wellbeing. Their review also includes studies on the relationship between teacher wellbeing and student achievement. The results show that, while some studies have found positive relationships between teacher wellbeing and student achievement, no studies have established causal links in these relationships; that is, no research has demonstrated that teacher wellbeing influences student achievement. Similarly, while some studies included in this review found a relationship between teacher wellbeing and school outcomes, they provided no evidence of causality.

An integrative review by Acton and Glasgow (2015) explored how teacher wellbeing is articulated, explained, and investigated. The authors identified three major themes in the research on teacher wellbeing: emotional work, professional relationships, and contextual factors. These themes were then compared to the literature on the effects of neoliberal policies on education. However, this review did not gather information on the potential impacts of teacher wellbeing on other factors.

Gray et al. (2017) explored the literature that had examined the relationships of several specific psychological concepts, such as mental health, resilience, and burnout, with teacher wellbeing. This review also analyzed how teacher wellbeing and the school climate might impact student learning. However, none of the included studies identified the causal effect patterns in the relationships of teacher wellbeing with the school climate or student learning.

The comprehensive review by McCallum et al. (2017) focused on the definitions of teacher wellbeing and on the factors that impact and initiatives that enable teacher wellbeing. Although the authors did not conduct searches on the impact of teacher wellbeing and did not present conclusive evidence on the matter, they argued that “teacher wellbeing is […] of critical relevance for whole school wellbeing and for students, but is also relevant for financial and economic considerations” (McCallum et al., 2017, p. 15).

In 2020, Viac and Fraser published an integrative research review that provides a framework for collecting the data and analyzing the research on teacher wellbeing. This work did not set out to report on every aspect of previous research but rather to present the findings and concepts that can be integrated into a single comprehensive framework. As such, the authors only briefly reported the findings related to the outcomes of teacher wellbeing. Instead, they framed the potential outcomes by distinguishing the internal outcomes of teacher wellbeing (i.e., stress, burnout, and motivation to leave the profession) from external outcomes (i.e., classroom processes and student wellbeing).

In the most recent review, Hascher and Waber (2021) collected and evaluated the studies on teacher wellbeing that were published from 2000 to 2019. Along with the concepts and measurement approaches, they investigated the potential outcomes of teacher wellbeing. However, this topic was only discussed briefly in one short paragraph, as it was not the main focus of their work. In this paragraph, the authors reported that most studies on this topic used cross-sectional or correlative methods, which might only hint at the potential outcomes.

In addition to the works with a strong focus on the concept of teacher wellbeing, some reviews investigated the impact of interventions to promote teacher wellbeing. For example, Naghieh et al. (2015) reviewed the literature on the effectiveness of organizational interventions for improving teacher wellbeing. Dreer and Gouasé (2021) reviewed the easy-to-use wellbeing interventions that can be implemented by individuals (rather than by an entire school). Vo and Allen (2022) presented a systematic review of the school-based interventions targeting teacher wellbeing. More recently, Beames et al. (2022) published a systematic review and meta-analysis of the intervention programs targeting mental health, professional burnout, and/or teacher wellbeing in schoolteachers. However, none of these reviews of intervention programs collected studies on the potential outcomes of teacher wellbeing.

Several research reviews have shed light on the topic of schoolteacher wellbeing. Most of them focused on the various understandings and conceptualizations of wellbeing; some examined the measurement approaches, while others investigated the effectiveness of targeted interventions for improving wellbeing. However, to date, no review has collected and evaluated the studies that have investigated the outcomes of teacher wellbeing.

## 3. Method

### 3.1. Objective and research questions

This review aims to add to the existing knowledge on teacher wellbeing and fill the gap in current research by evaluating studies that examine the potential outcomes of teacher wellbeing. It will address two research questions (RQs):

RQ 1: What are the characteristics of the reviewed studies investigating the potential outcomes of teacher wellbeing?
RQ 2: What evidence regarding the potential outcomes of teacher wellbeing can be gathered from previous research?

To address these questions, a systemic review was conducted following the preferred reporting items for systematic reviews and meta-analyses (PRISMA) guidelines (Moher et al., 2009); this review uses the PRISMA checklist (Page et al., 2021).

### 3.2. Identification sources and search strategy

The research on teacher wellbeing has become more diverse in recent years, with individual studies placing greater emphasis on specific sub-aspects. In certain instances, these studies may have used the name of a specific concept instead of the term “wellbeing” in the title. However, it is notable that the term “teacher wellbeing” has been consistently used in the abstracts and keywords of such works. Therefore, it seems appropriate to have focused the search primarily on the term “teacher wellbeing” (with variations in spelling) and to have adjusted the search criteria to include titles, abstracts, and keywords. From December 2022 until mid-February 2023, a literature search for peer-reviewed content and published doctoral dissertations was conducted on five major electronic databases (EBSCOhost, including PsycInfo and PsycArticle, Google Scholar, and ResearchGate). No specific time frame of publication was defined for the search and the following combination of search terms was used: “teacher” AND “wellbeing” OR “wellbeing” OR “teacher('s) wellbeing” OR “teacher('s) wellbeing” OR “wellbeing of teachers” OR “wellbeing of teachers” AND “outcomes” OR “benefits” OR “effects” OR “impact” or “effectiveness” OR “consequences” OR “merits” OR “associations.” In addition, the question “What are the effects of teacher wellbeing?” was fed to the elicit.org artificial intelligence research assistant. The search results were exported into CSV tables that were then merged into one table with the duplicate results removed. From this initial search, 397 entries were identified; these entries were then screened using the following criteria.

### 3.3. Selection criteria and data collection

The initial 397 papers were filtered by title, abstract, and keywords. As indicated by the PRISMA statement (Page et al., 2021), the eligibility criteria were used to include and exclude studies.

First, for studies to be included in the in-depth analysis, they had to be primary research published in peer-reviewed journals or accepted doctoral dissertations (c1: external quality assurance). Therefore, books (e.g., Hopman, 2021), book chapters (e.g., Olsen et al., 2021), and reports (e.g., Bajorek et al., 2014) were excluded. Second, the full text had to be written in English (c2: language). Third, publications that did not report any original data (c3: data), such as those that only reported concepts or frameworks (e.g., Lavy and Berkovich-Ohana, 2020), were excluded from the in-depth analysis. Fourth, the samples of the included studies consisted primarily of schoolteachers (c4: sample); therefore, the studies focusing on pre-school, nursery school, or student teachers (e.g., Hartl et al., 2022) were excluded. Fifth, as in previous reviews (Viac and Fraser, 2020; Hascher and Waber, 2021), the included studies had to have operationalized teacher wellbeing as a multidimensional construct that included at least one positive component in a set of two or more dimensions (c5: construct). Therefore, the studies that focused only on the negative aspects of teacher wellbeing, such as emotional exhaustion, were excluded (e.g., Carroll et al., 2021). Sixth, the included studies focused on the potential outcomes of teacher wellbeing (c6: outcomes); therefore, the studies investigating only the factors that influence wellbeing (e.g., Collie et al., 2021; Maas et al., 2022) were excluded.

**Figure 2:**
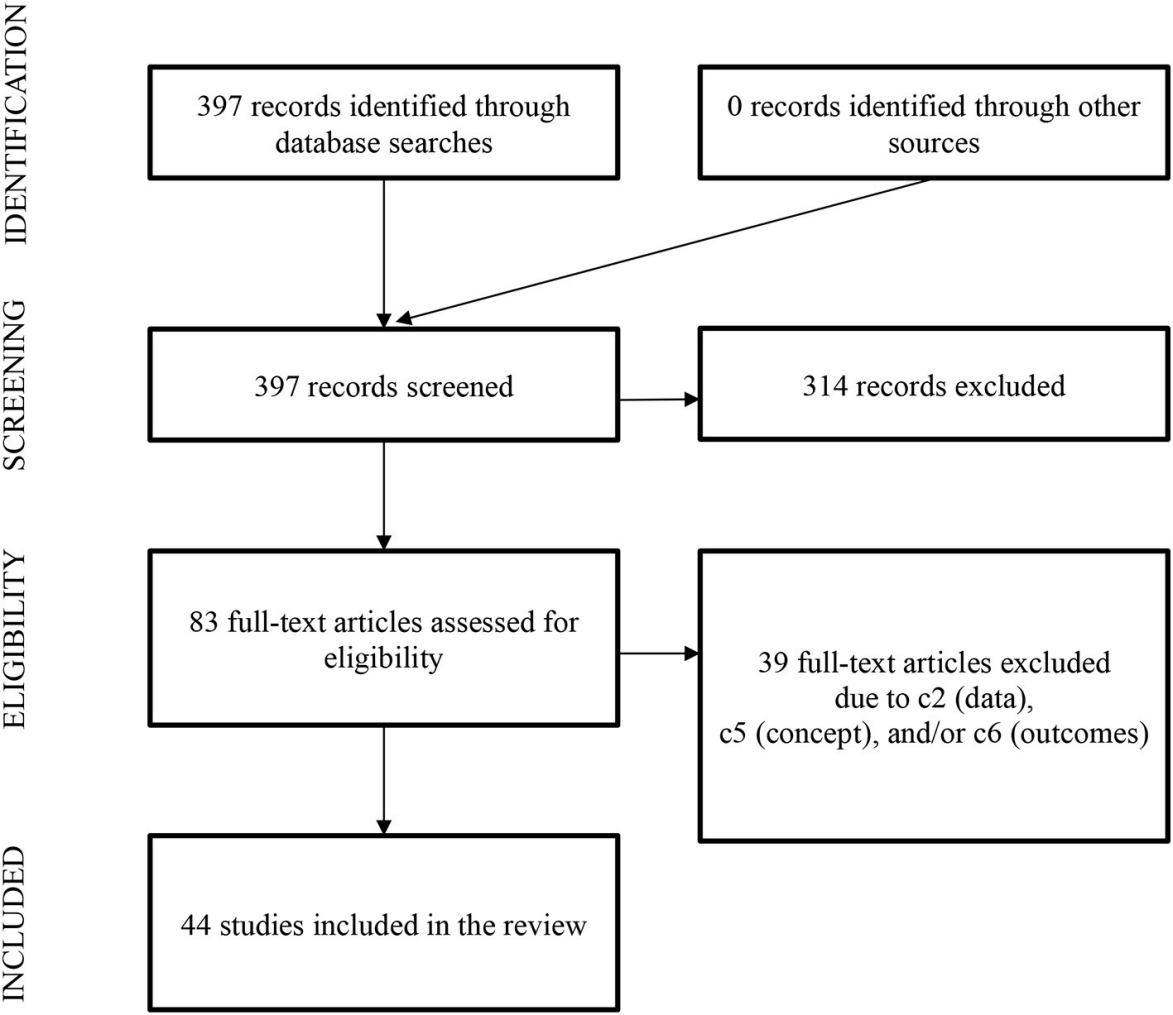
Flow chart of the screening procedure.

After the 397 research papers were screened using these criteria, 314 articles were excluded (see Figure 2). Most of the publications were excluded because they did not satisfy the criteria of c1 (quality assurance), c3 (data), or c4 (sample). The remaining 83 studies were reviewed in more depth. During this step, an additional 39 were excluded because they did not meet the selection criteria of c2 (data), c5 (concept), or c6 (outcomes; e.g., Tikkanen et al., 2021; Klusmann et al., 2022). Data were extracted from the remaining 44 papers; these data were then prepared for presentation.

### 3.4. Data extraction

The full texts of the 44 included studies were used to create the database for the following review. To provide a systematic overview, the key information necessary for unambiguously identifying the papers was collected along with the information relevant for answering the two research questions (see Table 1). Hence, the following information was extracted from the included studies: bibliographical information, contextual information (country, school type), information about the sample, the methodological approach (e.g., cross-sectional, longitudinal), method of data collection (e.g., questionnaire, interviews), operationalization of teacher wellbeing, potential outcomes investigated, and findings.

**Table 1:** Overview of reviewed studies.

**No**.	**References**	**Country/ countries**	***N***	**Sample**	**Type of data**	**Measurement approach**	**Method of data collection**	**Teacher wellbeing operationalization**	**Potential outcome (direction of relationship)**
1	Aldrup et al. (2018)	Germany	220 teachers 4,111 students	Secondary school teachers	Longitudinal	Quantitative	Questionnaires	Enthusiasm, exhaustion	Teacher–student relationships (+)
2	Billett et al. (2022)	Australia	532 teachers	Mixed sample	Cross-sectional	Quantitative	Online questionnaires	PERMA	Teacher self-efficacy (+)
3	Bilz et al. (2022)	Germany	805 teachers 2,686 students	Mixed sample	Cross-sectional	Quantitative	Questionnaires	Subjective psychological wellbeing, emotional exhaustion	• Student life satisfaction (+) • Student satisfaction with school (+) • Student subjective health complaints (-) • Student-perceived teacher support (+)
4	Brachfeld (2018)	USA	24 teachers 540 students	Elementary school teachers	Longitudinal	Quantitative qualitative	Questionnairesrecords	Self-efficacy, stress, emotional regulation, work–life balance	Office disciplinary referrals as proxy for classroom management (-)
5/6	Braun (2021) and Braun et al. (2020)	Canada, USA	15 teachers 320 students	Elementary school teachers	Longitudinal	Quantitative	Questionnaires	Life satisfaction, occupational burnout	Student-reported: positive outlook (+) emotional distress (-)
7	Burić and Moè (2020)	Croatia	536 teachers	High school teachers	Longitudinal	Quantitative	Questionnaires	Positive affect, job satisfaction	• Enthusiasm (+) • Self-efficacy (+)
8	Capone and Petrillo (2020)	Italy	285 high school teachers	High and middle school teachers	Cross-sectional	Quantitative	Questionnaires	Job satisfaction, burnout	• Self-efficacy (+) • Collective teacher efficacy (+)
9	Christian (2017)	USA	67 teachers	Elementary school	Longitudinal, randomized controlled trial	Quantitative qualitative	Questionnaires, observations	Subjective wellbeing, emotional exhaustion	Observed quality of teacher–student interactions (+)
10	Collie (2023)	Australia	426 teachers	Primary and secondary school teachers	Cross-sectional	Quantitative	Online questionnaires	Subjective vitality, behavioral engagement, professional growth	Turnover intentions (-)
11	Crain et al. (2017)	Canada USA	113 teachers		Longitudinal, randomized controlled trial	Quantitative	Questionnaires	Subjective wellbeing, mindfulness, job satisfaction	Quality of sleep (+)
12	Crider (2022)	USA	5 teachers	K-12 public school teachers	Longitudinal	Qualitative	Interviews	PERMA	Quality of instruction (+)
13	Dreer (2021)	Germany	511 teachers	Mixed sample	Cross-sectional	Quantitative	Online questionnaires	PERMA	Job satisfaction (+)
14	Duckworth et al. (2009)	USA	390 teachers	Mixed sample	Longitudinal	Quantitative	Questionnaires	Optimistic explanatory style, grit, life satisfaction	Teacher performance ranking by administrators (+)
15	Erickson (2021)	USA	86 teachers	Mixed sample	Cross-sectional	Quantitative	Questionnaires	Mindfulness practices	• Job satisfaction (+) • Self-efficacy (+) Resilience (+) • Burnout (-)
16	Glazzard and Rose (2020)	UK	35 teachers, 64 pupils	Primary school teachers	Cross-sectional	Qualitative	Interviews	Subjective wellbeing	Pupils' progress (+)
17	Granziera et al. (2023)	Australia	486 teachers	Elementary school teachers	Cross-sectional	Quantitative	Questionnaire	Behavioral engagement, emotional exhaustion	Student academic achievement (+)
18	Harding et al. (2019)	England and Wales	1,182 teachers 3,216 students	Secondary school teachers	Cross-sectional	Quantitative	Questionnaires	Subjective wellbeing, depressive symptoms	Student-reported: • Wellbeing (+) • Psychological distress (-)
19	Harrison et al. (2023)	48 countries	47,315 teachers	Mixed sample	Cross-sectional	Quantitative	Questionnaires	Job satisfaction	• Student–teacher relationships (+) • instructional quality (+)
20	Hofmann et al. (2022)	Germany	326 teachers	Elementary school teachers	Cross-sectional	Quantitative	Questionnaires	Positive and negative affect, mental health activities	Work behavior (+)
21	Huang et al. (2019)	China	1,115 teachers	Not stated	Cross-sectional	Quantitative	Questionnaires	Workplace wellbeing, anxiety, depression	Self-efficacy (+)
22	Jacobsson et al. (2016)	Sweden	521 teachers	Mixed sample	Cross-sectional	Quantitative	Questionnaires	Work satisfaction, emotional exhaustion	Teacher team effectiveness (+)
23	Jones Kingsley (2021)	USA	255 teachers	K-12 teachers	Cross-sectional	Quantitative	Questionnaires	PERMA	Teaching effectiveness (+)
24	Kern et al. (2014)	Australia	135 school employees	College teachers	Cross-sectional	Quantitative	Online questionnaires	PERMA	• Job satisfaction (+) • Life satisfaction (+) • Health/vitality (+)
25	Klusmann et al. (2008)	Germany	1,789 teachers	Secondary school teachers	Cross-sectional	Quantitative	Questionnaires	Job satisfaction, emotional exhaustion	• Student ratings of teachers' instructional behavior and classroom management (0) tempo (+) • cognitive activation (+) • perceived social support (+)
26	Kwon et al. (2022)	USA	667 teachers	Mixed sample	Cross-sectional	Quantitative	Questionnaire	Job commitment, depressive symptoms	Intent to leave (- with depressive symptoms only)
27	Lee et al. (2021)	USA	246 teachers	Secondary physical education teachers	Cross-sectional	Quantitative	Online questionnaire	Mindfulness, resilience, emotional exhaustion	Turnover intention (-)
28	Mead (2022)	Australia	11 teachers	Primary and secondary school teachers	Cross-sectional	Qualitative	Interviews	Health, feeling of value and social connection, sense of purpose and fulfillment	Teacher–student relationship (+)
29	Ostroff (1992)	USA	13,808 school staff	Senior and junior high school teachers	Cross-sectional	Quantitative	Questionnaires	Job satisfaction, commitment, stress	• Organizational performance (+) • Student satisfaction (+) • Teachers' intent to quit (-)
30	Pap et al. (2023)	Romania	66 teachers 410 students	High school and university teachers	Longitudinal	Quantitative	Questionnaires	Subjective wellbeing	Student-reported: • Student health (+) • Perceived teacher support (+)
31	Pfleging (2022)	USA	140 teachers	Secondary school teachers	Cross-sectional	Quantitative	Online questionnaires	Resilience, trait emotional, intelligence	Self-efficacy (+)
32	Pugliese (2019)	USA	80 teachers	Middle school teachers	Cross-sectional	Quantitative	Questionnaires	Subjective wellbeing	• Teachers' perceptions of bidirectional teacher–student relationships (+) • Students' perceptions of bidirectional teacher–student relationships (+)
33	Roffey (2012)	Australia	Not specified: 20–36 teachers, students and school counselors	Primary and high school teachers	Cross-sectional	Qualitative	Interviews	Staff wellbeing	Teacher–student relationship (+)
34	Sánchez Solarte (2022)	USA	203 L2 teachers 1,544 students	Secondary school teachers	Cross-sectional	Quantitative	Questionnaires	PERMA	Teacher-reported: • Positive relationships (+) • instructional delivery and learning • Classroom (+) Management and student behavior (+) • Use of assessment in instruction (+) Student-reported: • Learner engagement (0)
35	Sandilos et al. (2022)	USA	80 teachers	Elementary school teachers	Longitudinal, randomized controlled trial	Quantitative qualitative	Questionnaires, classroom observation	Emotional wellbeing	classroom organization in observed teacher–student interactions (+)
36	Shoshani (2021)	Israel	155 teachers	High school teachers	Cross-sectional data from a randomized controlled trial	Quantitative	Questionnaires	Job satisfaction, positive and negative affect	Teacher-reported: • Teacher emotional efficacy (+) • Student dropout rates (-) • Math grades (+)
37	Turner and Thielking (2019)	Australia	5 teachers	Mixed sample	Longitudinal	Qualitative	Interviews	PERMA	• Teaching behavior (+) • Student outcomes (+)
38	Turner et al. (2021)	Australia	119 teachers	Mixed sample	Cross-sectional	Quantitative	Online questionnaires	PERMA	• Teaching behavior (+) • Student outcomes (+)
39	Van Petegem et al. (2007)	Belgium	Number of teachers not specified; 433 students	Vocational training schools	Cross-sectional	Quantitative	Questionnaires	Wellbeing of the teacher measures	Teacher- and student-reported: • Teacher interactions (+) • Student wellbeing (+)
40	Virtanen et al. (2019)	Norway	79 teachers	Lower secondary teachers	Longitudinal	Quantitative qualitative	Questionnaires, classroom observation	Job satisfaction, emotional exhaustion	Observed classroom interactions: • Emotional support (+) • Classroom organization (+) • Instructional support (+)
41	Walter (2020)	USA	131 teachers	Mixed sample	Cross-sectional	Quantitative qualitative	Questionnaires, interviews	PERMA	• Intent to stay in the profession (+) • Teacher–student relationships (+)
42	Wang and Hall (2021)	Canada	1,086 teachers	Mixed sample	Longitudinal	Quantitative	Questionnaires	Teaching-related emotions, coping strategies	Intention to quit (-)
43	Wang et al. (2021)	Canada	1,086 teachers	Mixed sample	Longitudinal	Quantitative	Questionnaires	Job satisfaction, emotional exhaustion	Perceived student engagement (+)
44	Zheng et al. (2022)	China	1,821 teachers	Mixed sample	Cross-sectional	Quantitative	Questionnaires	Job satisfaction, burnout	• Teacher self-efficacy (+) • Teacher responsibility (+)

## 4. Results

### 4.1. Characteristics of the included studies (RQ 1)

#### 4.1.1. Core characteristics

**Figure 3:**
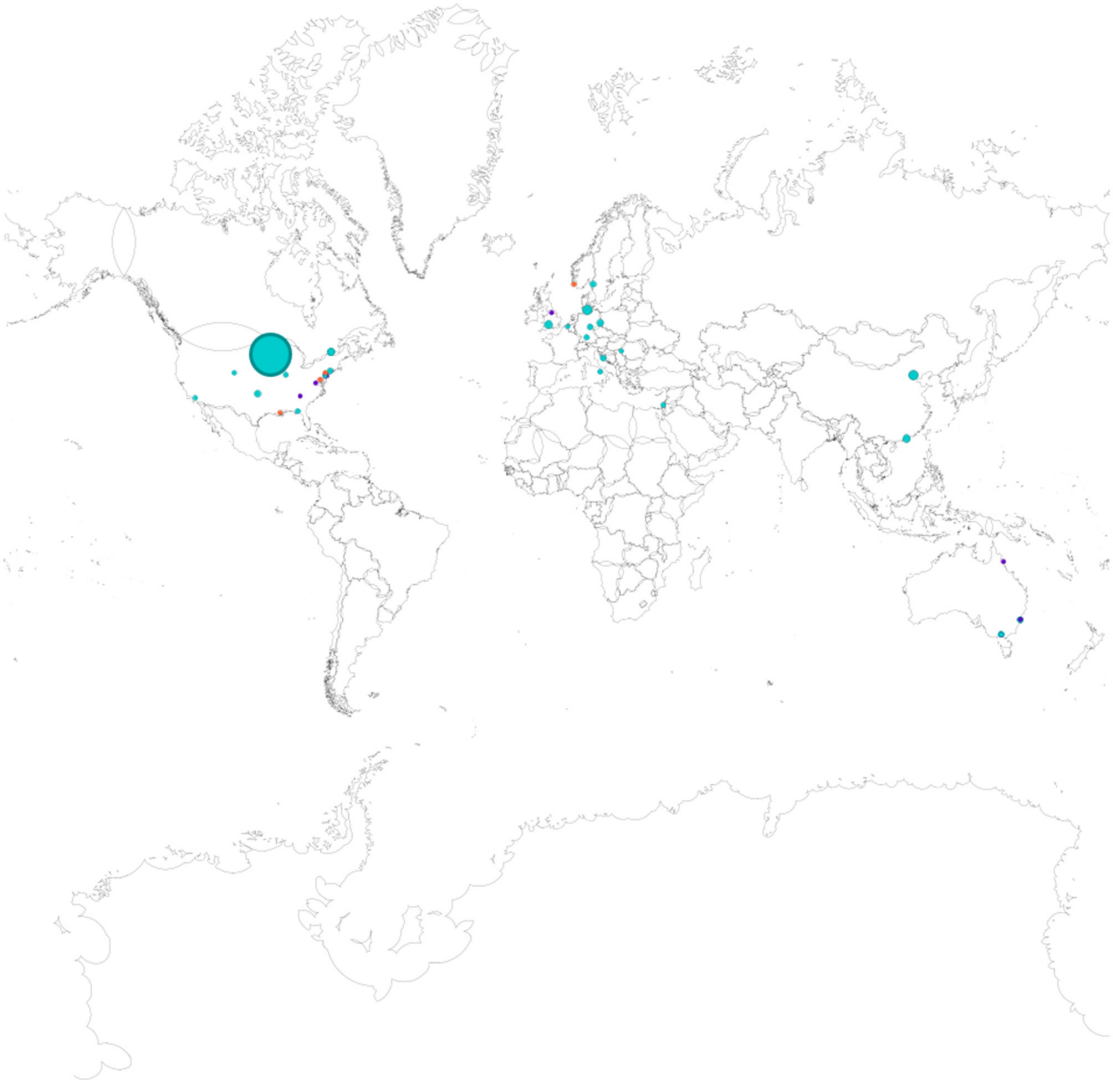
Geographic locations of the studies (node sizes are proportional to sample sizes; turquoise = quantitative studies; lilac = qualitative studies; orange = mixed-methods studies; study 19 is not shown here as the sample includes data from multiple countries).

A total of 44 studies were included in the in-depth review. To answer the first research question (RQ 1), key data were gathered from each research paper; these data are shown in Table 1. Most of the sources included in this review (*n* = 28) had been published in 2020 or later. The included studies were conducted in North America (19), Europe (14), Australia (8), and China (2). One study (no. 19) analyzed data collected in 48 different countries. In total, data were collected from more than 76,990 teachers, although some of the included studies did not specify the exact number of participating teachers. The teacher sample sizes in the reviewed studies range from five to 47,315 participants (see Table 1). Most of the studies (*n* = 32) included data collected from 100 to 1,200 teachers; nine studies used samples of fewer than 100 teachers, and three studies included data from samples of over 1,200 teachers. Figure 3 provides an overview of the geographic locations and sample sizes of the studies. From this figure, it can be seen that the majority of the studies on the outcomes of teacher wellbeing were conducted in North America or Europe. The figure also shows that Asia and Australia are underrepresented, and, as yet, no (published) studies on this topic have been conducted in South America or Africa. Moreover, the sizes of the nodes illustrate that the studies with larger sample sizes were conducted in the United States, Europe, and China. Unsurprisingly, the studies that used qualitative (lilac) or mixed-methods (orange) approaches have the smallest sample sizes.

#### 4.1.2. Conceptualizations and measures of teacher wellbeing

The examination of theoretical concepts applied in the reviewed studies investigating teacher wellbeing revealed the presence of four approaches. Among these approaches, the concept of subjective wellbeing (Diener, 2000) emerged as prevalent, appearing in 19 studies. This concept entails a multidimensional construct encompassing positive/negative dimensions and emotional/cognitive factors. To assess this construct, researchers commonly employed scales that measured job satisfaction and emotional exhaustion/burnout (e.g., studies 8, 25, and 29). Because most of the studies relied on well-tested and established measures, the scales generally showed acceptable reliability. For example, Cronbach's alpha for job satisfaction ranged between 0.70 and 0.90 across the studies.

Positive psychology served as the foundation for the wellbeing conceptualization in 11 studies. One study explored the feelings of value, social connection, sense of purpose, and fulfillment, while the remaining studies in this category adopted the PERMA model of wellbeing (Seligman, 2011). The model identifies five essential components of wellbeing, that is, positive emotions (P), engagement (E), relationships (R), and meaning (M). Across the majority of the PERMA-focused studies (e.g., studies 2, 13, and 23), the model was operationalized using versions of the PERMA profiler (Butler and Kern, 2016). The reliability of these measures was generally satisfactory, with Cronbach's alpha coefficients ranging from 0.62 to 0.88 across the different subscales and studies. One study (Study 2) did not report alpha values. In other studies (e.g., study 24), the PERMA building blocks were measured using independent scales from different sources. Moreover, PERMA was used as a theoretical framework when analyzing the qualitative data (e.g., Studies 12 and 37).

In the third category, seven studies addressed wellbeing using behavior-oriented concepts from interdisciplinary theoretical backgrounds, for example, the studies focused on optimistic explanatory style (Study 14), behavioral engagement (Study 17), mindfulness practices (Studies 15, 27), or job commitment (Study 26). The applied measures included psychometric mindfulness and resilience scales (e.g., Study 27) showing acceptable reliability with Cronbach's alpha ranging between 0.87 and 0.92.

A smaller subset of the studies (4) focused on teacher wellbeing concerning emotions. Examples include trait emotional intelligence (Study 31), emotional wellbeing (Study 35), and teaching-related emotions (Study 42). Here, the assessment of wellbeing mainly relied on scales measuring the state and trait emotions of teachers. Again, the reliability of the applied measurement scales can be described as acceptable, with Cronbach's alpha ranging between 0.76 and 0.91 across the studies.

The smallest subset of studies (3) applied unique approaches to conceptualize and measure the staff wellbeing and teacher wellbeing (Studies 19, 33, and 39). In these cases, the assessments relied on the combined measurements of teacher satisfaction and self-efficacy scales (Studies 19, 39) or interviews (Study 33).

#### 4.1.3. Applied methods

As shown in Table 1, most of the reviewed articles used a quantitative approach (*n* = 34), followed by qualitative approaches (*n* = 5) and mixed-methods designs (*n* = 5). Most of the studies (*n* = 41) collected data using questionnaires; only three studies relied exclusively on interviews. Three studies collected data using both interviews and questionnaires. As Figure 3 shows, there seems to be no correlation between the location of the study and the use of a qualitative (lilac) or mixed-methods (orange) approach. Most of the included studies used cross-sectional data (*n* = 30); fewer included longitudinal data (*n* = 14). Of those that included longitudinal data, only three studies described randomized controlled trials (Studies 9, 11, and 35), even though this approach sets a high standard in causality research. Interestingly, longitudinal data were also used in some qualitative and mixed-methods studies (e.g., Studies 4 and 12).

In the qualitative (Studies 12, 16, 28, 33, and 37) and mixed method approaches (Studies 4, 9, 35, 40, and 41), semi-structured interviews were the prevailing method in six studies (Studies 12, 16, 28, 33, 37, and 41). Classroom observations were used in three cases (Studies 9, 35, and 40). These studies relied on different structured observation tools rating the behaviors in teacher-student interactions. Document analysis was used in one approach (Study 4), which included the data from disciplinary office referrals as a proximal indicator of classroom management.

The reviewed studies included samples of the teachers who worked at various types of schools, including primary/elementary schools, secondary or middle schools, high schools, and vocational training schools. Some of the studies included teachers from more than one type of school (mixed sample), and some did not report the type(s) of school in the description of the sample (e.g., Study 21). Ten studies included students or other school staff and teachers in the sample (e.g., Studies 1–6).

The assessment of the potential outcomes covers many of the aspects in the theoretical spectrum (see Figure 1). Table 2 shows the distributions of studies within this spectrum. While, again, most of the studies used questionnaires to assess the outcomes, some studies also incorporated additional material, such as grades or referral records. However, only 11 of the 44 included studies used student data to capture the potential effects of teacher wellbeing.

**Table 2:** Reviewed studies sorted by type of evidence and factors within the spectrum of potential outcomes.

**Potential outcomes**	**Exploratory evidence**	**Correlational evidence**	**Causal evidence**
Health and sleep quality	Kern et al. (2014) and Burić et al. (2021)		Crain et al. (2017)
Job and life satisfaction		Silvia et al. (2012); Kern et al. (2014); Butler and Kern (2016); Capone and Petrillo (2020); Collie et al. (2021); Dreer (2021); Erickson (2021)	
Motivation to quit		Ostroff (1992); Walter (2020); Lee et al. (2021); Kwon et al. (2022); Collie (2023)	Wang and Hall (2021)
Job engagement		Ostroff (1992); Silvia et al. (2012); Kern et al. (2014); Shoshani (2021); Hofmann et al. (2022); Collie (2023)	
Self-efficacy		Huang et al. (2019); Capone and Petrillo (2020); Erickson (2021); Billett et al. (2022); Pfleging (2022); Zheng et al. (2022)	Burić et al. (2021)
Teacher–student relationships	Roffey (2012); Pugliese (2019); Walter (2020)	Christian (2017); Aldrup et al. (2018); Pugliese (2019)	
Teamwork		Jacobsson et al. (2016)	
Choice of teaching methods		Huang et al. (2019)	
Teaching behavior	Turner and Thielking (2019); Turner et al. (2021)	Huang et al. (2019)	Ryan and Deci (2016)
Quality of instruction	Klusmann et al. (2008); Crider (2022)	Klusmann et al. (2008); Silvia et al. (2012); Brachfeld (2018); Virtanen et al. (2019); Jones Kingsley (2021); Sánchez Solarte (2022); Harrison et al. (2023); Pap et al. (2023)	
Student experience	Van Petegem et al. (2007); Turner and Thielking (2019); Glazzard and Rose (2020); Turner et al. (2021)	Ostroff (1992); Harding et al. (2019); Bilz et al. (2022); Pap et al. (2023)	Braun et al. (2020); Braun (2021)
Student outcomes	Turner et al. (2021)	Huang et al. (2019); Shoshani (2021)	Duckworth et al. (2009); Braun et al. (2020)
Student engagement		Sánchez Solarte (2022)	Wang and Hall (2021)
School outcomes		Ostroff (1992); Granziera et al. (2023)	
System outcomes			

### 4.2. Evidence for the potential outcomes of teacher wellbeing (RQ 2)

#### 4.2.1. Assessment of outcomes

The reviewed studies utilized various outcome measures that encompassed a wide range of potential teacher wellbeing outcomes (see Figure 1). In line with measuring teacher wellbeing, most of the studies employed self-report measures in the form of questionnaires to assess the potential outcomes. Some examples of the teachers' self-reports include evaluations of self-efficacy (e.g., Studies 7, 8, 15, 21, and 44), student-teacher relationships (Study 34), and their intentions to quit or stay in the profession (Studies 41 and 42). In addition, some studies incorporated student data, such as self-reported wellbeing (e.g., Studies 3, 5, 6, and 18), perceptions of classroom management, and teacher support (e.g., Studies 25 and 30). A smaller number of the studies involved self-report data from both teachers and students (Studies 32, 34, and 39). In other cases, the interactions between the students and teachers were recorded by third parties using observation methods (Studies 9, 35, and 40). Another approach (Study 4) utilized the records of disciplinary office referrals as an indicator of teacher classroom management.

#### 4.2.2. Findings and classification

To address the second research question (RQ 2), the findings of the collected studies were classified into three categories: exploratory evidence, correlational evidence, and causal evidence. These categories were selected, as they align with scientific practices in knowledge generation (Bryman, 2006). The exploratory evidence consisted of studies that primarily employed qualitative approaches and smaller sample sizes and aimed at exploring the field, identifying patterns, and generating hypotheses. The studies in the second category provided correlational evidence often involving correlation analyses of the cross-sectional data. Such of the studies were typically designed to investigate and substantiate findings from exploratory research by testing hypotheses and establishing relationships and patterns. The studies in the third category provided causal evidence, often utilizing larger sample sizes and investigating the causal relationships between the variables using longitudinal data and/or experiments. These studies were specifically designed to test the causal hypotheses and examine the causal effects.

As indicated in Table 2, the majority of the studies (*n* = 34) provided correlational evidence regarding the relationship between teacher wellbeing and the potential outcome variables. Only a few studies (*n* = 9) offered exploratory evidence, primarily from qualitative research.

Even fewer offered causal evidence based on longitudinal studies and panel analyses or randomized controlled trials (*n* = 8). A few studies provided evidence in two of these categories (see Table 2).

Most of the reviewed works provided evidence highlighting the importance of teacher wellbeing. The qualitative studies offered exploratory evidence suggesting that teacher wellbeing is linked to teacher–student relationships, teaching behavior, quality of instruction, student experiences, and student outcomes. Most of the reviewed quantitative studies found positive correlations between teacher wellbeing and desired outcomes across the entire spectrum of potential outcomes (e.g., teacher self-efficacy, teacher behavior, quality of instruction, student wellbeing, and school outcomes). Conversely, (positively valenced) teacher wellbeing has been found to be negatively related to unwanted outcomes (e.g., teachers' turnover intentions, students' health complaints). Interestingly, only a few studies reported null findings among the investigated relationships (e.g., Klusmann et al., 2008). Most of the correlations found in the studies are statistically significant and range from small to large effect sizes; the majority of the studies reported medium correlations.

This review has identified only eight studies that used more advanced approaches to investigate the outcomes of teacher wellbeing and included longitudinal data in their analyses. These works indicated that teacher wellbeing positively influences teachers' quality of sleep (Crain et al., 2017), intention to stay in the job (Wang and Hall, 2021), perceived student engagement (Wang et al., 2021), enthusiasm and self-efficacy (Burić and Moè, 2020), and behavior (Sandilos et al., 2022), as well as the students' experiences in the classroom and student outcomes (Duckworth et al., 2009; Braun et al., 2020; Braun, 2021). The results of the causality analyses conducted by Pap et al. (2023) are somewhat inconclusive. While the authors discovered large initial correlations between teacher wellbeing and the students' perceptions of teacher support, these associations were no longer significant when students' educational level was also included in the prediction.

## 5. Discussion

Almost every publication on teacher wellbeing has highlighted the positive implications of wellbeing or warned of the drastic consequences of declining teacher wellbeing (Klusmann and Waschke, 2018). To date, however, no solid evidence to support such claims has been systematically gathered. The results of this systematic review of 44 studies addressing two research questions suggest that the potential outcomes of teacher wellbeing have received increased attention in recent years. Most of the included studies were published during only the last 3 years and could, therefore, not have been included in previous reviews (Viac and Fraser, 2020; Hascher and Waber, 2021).

### 5.1. Prevalent findings

Like those in previous reviews, most of the included studies on the potential outcomes of teacher wellbeing found correlative relationships between teacher wellbeing and the various examined outcomes. In line with previous reviews, the present paper also finds reports of such relationships for a broad spectrum of possible outcomes, from individual benefits to systemic benefits for students and schools. Another novel insight of this review is that these correlational findings appear to be consistent across the different countries where the included studies were conducted. This suggests that teacher wellbeing plays an important role in the interplay of several factors highly relevant to educational success. However, scholarly attention and evidence on this issue are not equally distributed across the spectrum of possible outcomes. For example, while a number of studies addressed teacher engagement, only one included study examined the relationship of teacher wellbeing with teamwork in schools. Moreover, evidence is scarce regarding the choice of teaching methods, a potential outcome of teacher wellbeing identified by some theoretical approaches (Frenzel, 2014), and none of the included studies investigated the possible impacts of teacher wellbeing on school systems, a connection suggested in previous literature (e.g., Bajorek et al., 2014; McCallum et al., 2017). Future research should address these gaps and investigate the spectrum of potential effects more systematically.

### 5.2. Directions of future research

Qualitative research designs could be especially useful for uncovering the previously unknown outcomes of teacher wellbeing and could prepare the ground in less-explored areas of research (e.g., effects on school systems). However, this review has identified only a few qualitative studies on the outcomes of teacher wellbeing. While this reflects the overall lack of qualitative research on teacher wellbeing (Bricheno et al., 2009; McCallum and Price, 2016; Hascher and Waber, 2021), it profoundly limits our understanding of the outcomes of teacher wellbeing. Therefore, more qualitative and mixed-methods studies on this topic are needed, especially in the areas where such research can offer additional perspectives and new sources of data.

Most of the studies included in this review primarily relied on teachers' self-reports. A minimal number of studies integrated self-report data from both teachers and students or conducted analyses based on the observations of teacher-student interactions. Notably, only one study within the entire sample adopted a distinctive approach by utilizing the records of disciplinary office referrals as an indicator of teacher classroom management. Despite its inherent limitations, this approach provides a valuable demonstration of employing a more objective method to document the outcomes pertaining to teacher wellbeing. The discovered prevalence of teacher self-reports in teacher wellbeing research is in line with previous findings and the need to incorporate the perspectives of other stakeholders, such as students, parents, and school administrators, in such research has been articulated before (Hascher and Waber, 2021). The findings from this review further emphasize the importance of the need for considering multiple perspectives. Relying solely on the self-reports of teachers appears especially insufficient when examining wellbeing outcomes.

In addition, more research is needed to investigate the actual causal links between teacher wellbeing and its potential outcomes. As demonstrated by the findings of Pap et al. (2023), even high correlations are no guarantee that a causality test will indicate a plausible causal connection. As this review has shown, only eight works used robust designs and presented results that demonstrate the impacts of teacher wellbeing. Randomized controlled trials in which a subsample receives an intervention that promotes teacher wellbeing can be used to assess the potential impacts of wellbeing, but few such studies have been published. Such studies, as well as other complex designs such as cross-lagged panel designs, would contribute to the existing knowledge in this area, as demonstrated by some studies in this review (e.g., Wang and Hall, 2021).

Apart from these overarching issues, future research should also reflect on the characteristics of previous works. The results of this review show that most existing research on the outcomes of teacher wellbeing has been conducted in North America or Europe. Conversely, this collection of papers contains no independent research conducted with teacher samples from South America or Africa, and only a few studies have been conducted in Asia or Australia. However, such research is essential to improving our understanding of the impact of cultural differences on the outcomes of teacher wellbeing, a need that was highlighted by Harrison et al. (2023).

In line with previous reviews, this review finds that studies on this topic use a wide range of conceptual understandings of wellbeing. As has been previously demonstrated (e.g., Hascher and Waber, 2021), most research on teacher wellbeing has used either the concept of subjective wellbeing (Diener, 2000) or the PERMA model of wellbeing (Seligman, 2011). However, there are other approaches to this concept; some focus on the behavioral aspects of wellbeing (e.g., mindfulness practices), while some focus primarily on emotions (e.g., emotional wellbeing, emotional regulation). Some of these approaches have been specifically targeted in previous reviews of this area (e.g., Gray et al., 2017). Overall, there is a lack of clarity in conceptions of wellbeing. For example, in some studies, teacher job satisfaction has been considered an indicator of subjective wellbeing (e.g., Burić and Moè, 2020); however, in other cases, it is defined as an outcome of teacher wellbeing (e.g., Dreer, 2021). Although these varied approaches enrich our understanding of the potential effects, they also hinder efforts to compare and integrate the results of various studies. To encourage systematic progress in the field, calls for a more consistent use of concepts (Dreer and Gouasé, 2021) in conjunction with multi-source assessments (Hascher and Waber, 2021) can only be reiterated.

## 6. Limitations

This review has some limitations that should be carefully considered when interpreting the findings. First, one limitation of this review is that it relied on the global search term “teacher wellbeing.” As a result, there is a possibility that studies exploring the sub-concepts of teacher wellbeing were missed, particularly those that did not explicitly use the term in their keywords or abstracts. Examples could involve concepts from positive psychology, like positive psychological capital (e.g., Zewude and Hercz, 2022) or from self-determination theory, like basic need fulfillment (e.g., Ryan and Deci, 2016). An illustration of this is seen in the study conducted by Moè and Katz (2022), who found a positive relationship between teachers' need satisfaction and teaching styles. To enhance the scope of future reviews, it could be beneficial to compile a semantic network of teacher wellbeing related terms that would allow for a broader search focus and capture a wider range of relevant studies. Next, the inclusion and exclusion criteria used in this review pose some limitations. Because this work includes only peer-reviewed articles and accepted dissertations (c1: external quality assurance), important findings published in book chapters, books, reports, or policy papers may have been excluded. As Hascher and Waber (2021) noted, this might be problematic, because these types of publications offer potential outlets for inconclusive or confusing findings that are, as a result, systematically excluded from the present review. Furthermore, by including only papers written in English (c2: language), this review might have overlooked solid and important evidence published in other languages. This criterion might also explain the lack of studies from certain geographic regions, but it also reinforces a practice that helps perpetuate the cultural bias that is already present within the publication system (e.g., Yousefi-Nooraie et al., 2006). As only studies that use samples consisting primarily of schoolteachers (c4: sample) are included here, studies conducted in the context of preschool education have been excluded. In addition, by excluding studies that did not operationalize teacher wellbeing as a multidimensional construct (c5: construct) with at least one positive component in a set of two or more dimensions, studies that focused on the negative aspects of teacher wellbeing, such as emotional exhaustion, were excluded. While a multidimensional concept might be the standard for current research on teacher wellbeing, this criterion might have also led to the exclusion of potentially interesting and important publications. However, this must be considered a small limitation, given that there are comprehensive reviews that focused on the effects of the negative elements of teacher wellbeing, such as, for example, the effects of teacher burnout (e.g., Madigan and Kim, 2021).

Second, like all literature reviews, the results of this review draw only on published research. It has been observed in many fields that the majority of studies included in reviews have at least some significant results to report; conversely, this suggests that non-significant results are not reported at all. Consequently, a general publication bias could mean that every review provides only a one-sided picture of the actual knowledge base on a subject (Kennedy, 2004).

## 7. Recommendations

In light of the findings of this systematic review, three important recommendations for the future development of research on teacher wellbeing are offered here.

First, more research needs to be done on the outcomes of teacher wellbeing. Most of the reviewed studies found that teacher wellbeing is related to important aspects of teachers' work lives, students' experiences and outcomes, and school outcomes. Therefore, it is now the duty of the research community to investigate the nature of these relationships. However, conducting research in schools, especially research that involves student data, is complex and requires careful planning, extensive recruiting, and (often) comprehensive permission requests. In view of these challenges, more funding and grants should be made available to support future research on this important topic. In particular, such funding sources should target researchers in countries that are not yet contributing to this discourse.

Second, this review highlights the fact that, while theoretical frameworks identify an entire spectrum of the potential outcomes of teacher wellbeing, recent research has not yet explored all of these aspects. Moreover, there might be even more potential outcomes that need to be discovered and added to this spectrum. Therefore, future research should aim to identify blind spots and systematically investigate the spectrum of possible outcomes. For example, there might be other theoretical frameworks that could help identify further potential outcomes. However, when using such an approach, researchers should avoid viewing this spectrum as a one-dimensional approach to the benefits of teacher wellbeing. The factors on the spectrum are interrelated, and the (dynamic) relationships should be investigated by examining several factors simultaneously.

Third, more research is needed to identify the causal links between teacher wellbeing and its potential outcomes. To do this, future studies must incorporate complex methods of data collection and analysis, of which cross-lagged panel designs and randomized controlled trails might only be examples. Researchers are encouraged to implement such approaches to help build a more solid knowledge base on this important matter.

## Data availability statement

The original contributions presented in the study are included in the article/supplementary material, further inquiries can be directed to the corresponding author.

## Author contributions

The author confirms being the sole contributor of this work and has approved it for publication.

## References

[B1] Acton R. Glasgow P. (2015). Teacher wellbeing in neoliberal contexts: a review of the literature. Austral. J. Teacher Educ. 40, 6. 10.14221/ajte.2015v40n8.6

[B2] Aldrup K. Klusmann U. Lüdtke O. Göllner R. Trautwein U. (2018). Student misbehavior and teacher well-being: testing the mediating role of the teacher–student relationship. Learn. Instr. 58, 126–136. 10.1016/j.learninstruc.2018.05.006

[B3] Bajorek Z. Gulliford J. Taskila T. (2014). Healthy Teachers, Higher Marks? Establishing a Link Between Teacher Health and Wellbeing and Student Outcomes. London: The Work Foundation.

[B4] Bakker A. B. Demerouti E. Euwema M. C. (2005). Job resources buffer the impact of job demands on burnout. J. Occup. Health Psychol. 10, 170–180. 10.1037/1076-8998.10.2.170 15826226

[B5] Beames J. Spanos S. Roberts A. McGillivray L. Li S. Newby J. . (2022). Intervention programs targeting the mental health, professional burnout, and/or wellbeing of school teachers: a systematic review and meta-analyses. Educ. Psychol. Rev. 2022, 1386793. 10.21203/rs.3.rs-1386793/v1 36876289 PMC9974401

[B6] Becker E. S. Goetz T. Morger V. Ranellucci J. (2014). The importance of teachers' emotions and instructional behavior for their students' emotions—An experience sampling analysis. Teach. Teacher Educ. 43, 15–26. 10.1016/j.tate.2014.05.002

[B7] Billett P. Turner K. Li X. (2022). Australian teacher stress, well-being, self-efficacy, and safety during the COVID-19 pandemic. Psychol. Schools 2022, 1–21. 10.1002/pits.22713 35942390 PMC9348030

[B8] Bilz L. Fischer S. M. Hoppe-Herfurth A.-C. John N. (2022). A consequential partnership: the association between teachers' well-being and students' well-being and the role of teacher support as a mediator. Zeitschrift für Psychologie 230, 264–275. 10.1027/2151-2604/a000497

[B9] Brachfeld M. C. (2018). The Relationship Between Teacher Well-being and the Use of Office Discipline Referrals in an Urban Charter School. (Doctoral dissertation), ProQuest Dissertations Publishing, Tulane University, New Orleans, LA, United States.

[B10] Braun S. E. (2021). Teachers' Occupational Health and Well-being: Antecedents, Consequences, and Prospects for Change. (Doctoral dissertation), ProQuest Dissertations Publishing, Pennsylvania State University, State College, PA, United States.

[B11] Braun S. S. Schonert-Reichl K. A. Roeser R. W. (2020). Effects of teachers' emotion regulation, burnout, and life satisfaction on student well-being. J. Appl. Dev. Psychol. 69, 101151. 10.1016/j.appdev.2020.101151

[B12] Bricheno P. Brown S. Lubansky R. (2009). Teacher Wellbeing: A Review of the Evidence. London: Teacher Support Network Research Services.

[B13] Bryman A. (2006). Integrating quantitative and qualitative research: how is it done? Qualit. Res. 6, 97–113. 10.1177/1468794106058877

[B14] Burić I. Kim L. E. Hodis F. (2021). Emotional labor profiles among teachers: Associations with positive affective, motivational, and well-being factors. J. Educ. Psychol. 113, 1227–1243. 10.1037/edu0000654

[B15] Burić I. Moè A. (2020). What makes teachers enthusiastic: the interplay of positive affect, self-efficacy and job satisfaction. Teach. Teacher Educ. 89, 103008. 10.1016/j.tate.2019.103008

[B16] Butler J. Kern M. L. (2016). The PERMA-Profiler: a brief multidimensional measure of flourishing. Int. J. Wellbeing 6, 1–48. 10.5502/ijw.v6i3.526

[B17] Cano S. L. Flores B. B. Claeys L. Sass D. A. (2017). “Consequences of educator stress on turnover: the case of charter schools,” in Educator Stress: An Occupational Health Perspective, eds T. M. McIntyre, S. E. McIntyre, and D. J. Francis (Berlin: Springer), 127–156. 10.1007/978-3-319-53053-6_6

[B18] Capone V. Petrillo G. (2020). Mental health in teachers: relationships with job satisfaction, efficacy beliefs, burnout and depression. Curr. Psychol. 39, 1757–1766. 10.1007/s12144-018-9878-7

[B19] Carolan S. Harris P. R. Cavanagh K. (2017). Improving employee well-being and effectiveness: systematic review and meta-analysis of web-based psychological interventions delivered in the workplace. J. Med. Internet Res. 19, e271. 10.2196/jmir.7583 28747293 PMC5550734

[B20] Carroll A. York A. Fynes-Clinton S. Sanders-Oonnor E. Flynn L. Bower J. . (2021). The downstream effects of teacher well-being programs: improvements in teachers' stress, cognition and well-being benefit their students. Front. Psychol. 12, 689628. 10.3389/fpsyg.2021.689628 34276519 PMC8281271

[B21] Christian E. M. (2017). The Effectiveness of the Achiever Adult Resilience Curriculum in Promoting Teacher Wellbeing. (Doctoral dissertation), ProQuest Dissertations Publishing, University of Minnesota, Minneapolis, MN, United States.

[B22] Collie R. J. (2023). Teacher well-being and turnover intentions: investigating the roles of job resources and job demands. Br. J. Educ. Psychol. 2023, 1–14. 10.1111/bjep.12587 36720462

[B23] Collie R. J. Martin A. J. Morin A. J. S. Malmberg L.-E. Sammons P. (2021). A multilevel person-centered examination of teachers' workplace experiences: replication and extension with links to instructional support and achievement. Front. Psychol. 12, 711173. 10.3389/fpsyg.2021.711173 34421763 PMC8377360

[B24] Crain T. L. Schonert-Reichl K. A. Roeser R. W. (2017). Cultivating teacher mindfulness: effects of a randomized controlled trial on work, home, and sleep outcomes. J. Occup. Health Psychol. 22, 138–152. 10.1037/ocp0000043 27182765

[B25] Crider J. S. (2022). Teachers' Experiences With Positive Emotion, Engagement, Relationships, Meaning, and Achievement (PERMA): A Qualitative Interview Study. (Doctoral dissertation), ProQuest Dissertations Publishing, Piedmont University, Athens, GA, United States.

[B26] Demerouti E. Bakker A. B. Nachreiner F. Schaufeli W. B. (2001). The job demands–resources model of burnout. J. Appl. Psychol. 86, 499–512. 10.1037/0021-9010.86.3.499 11419809

[B27] Diener E. (2000). Subjective well-being: the science of happiness and a proposal for a national index. Am. Psychol. 55, 34–43. 10.1037/0003-066X.55.1.34 11392863

[B28] Dreer B. (2021). Teachers' well-being and job satisfaction: the important role of positive emotions in the workplace. Educ. Stud. 2021, 1–17. 10.1080/03055698.2021.194087231380445

[B29] Dreer B. Gouasé N. (2021). Interventions fostering well-being of schoolteachers: a review of research. Oxf. Rev. Educ. 2021, 1–19. 10.1080/03054985.2021.2002290

[B30] Duckworth A. Quinn P. D. Seligman M. E. (2009). Positive predictors of teacher effectiveness. J. Posit. Psychol. 4, 540–547. 10.1080/17439760903157232

[B31] Erickson J. (2021). Mindfulness and Workplace Outcomes for Secondary Teachers. ProQuest Dissertations Publishing, The Claremont Graduate University, Claremont, CA, United States.27182765

[B32] Fredrickson B. L. (2001). The role of positive emotions in positive psychology: the broaden-and-build-theory of positive emotions. Am. Psychol. 56, 218–226. 10.1037/0003-066X.56.3.218 11315248 PMC3122271

[B33] Frenzel A. C. (2014). “Teacher emotions,” in International Handbook of Emotions in Education, eds A. Linnenbrink-Garcia and R. Pekrun (London: Routledge), 494–519.

[B34] Glazzard J. Rose A. (2020). The impact of teacher well-being and mental health on pupil progress in primary schools. J. Public Mental Health 19, 349–357. 10.1108/JPMH-02-2019-0023

[B35] Granziera H. Martin A. J. Collie R. J. (2023). Teacher well-being and student achievement: a multilevel analysis. Soc. Psychol. Educ. 22, 1. 10.1007/s11218-022-09751-130544017

[B36] Gray C. Wilcox G. Nordstokke D. (2017). Teacher mental health, school climate, inclusive education and student learning: a review. Can. Psychol. 58, 203–210. 10.1037/cap0000117

[B37] Harding S. Morris R. Gunnell D. Ford T. Hollingworth W. Tilling K. . (2019). Is teachers' mental health and wellbeing associated with students' mental health and wellbeing? J. Affect. Disord. 253, 460–466. 10.1016/j.jad.2019.03.046 30189355

[B38] Harrison M. G. King R. B. Wang H. (2023). Satisfied teachers are good teachers: the association between teacher job satisfaction and instructional quality. Br. Educ. Res. J. 2023, 3851. 10.1002/berj.3851

[B39] Hartl A. Holzberger D. Hugo J. Wolf K. Kunter M. (2022). Promoting student teachers' well-being: a multi-study approach investigating the longitudinal relationship between emotional exhaustion, emotional support, and the intentions of dropping out of university. Zeitschrift für Psychologie 230, 241–252. 10.1027/2151-2604/a000495

[B40] Hascher T. Waber J. (2021). Teacher well-being: a systematic review of the research literature from the years 2000–2019. Educ. Res. Rev. 34, 100411. 10.1016/j.edurev.2021.100411

[B41] Hatfield E. Cacioppo J. T. Rapson R. L. (1993). Emotional Contagion. Curr. Direct. Psychol. Sci. 2, 96–100. 10.1111/1467-8721.ep10770953

[B42] Hofmann H. Groß D. Kohlmann C.-W. (2022). On the role of mental health activities for teachers' work and life. Appl. Res. Qual. Life 17, 205–227. 10.1007/s11482-020-09885-4

[B43] Hopman J. (2021). Surviving Emotional Work for Teachers: Improving Wellbeing and Professional Learning Through Reflexive Practice. London: Routledge/Taylor and Francis Group.

[B44] Houser M. L. Waldbuesser C. (2017). Emotional contagion in the classroom: the impact of teacher satisfaction and confirmation on perceptions of student nonverbal classroom behavior. Coll. Teach. 65, 1–8. 10.1080/87567555.2016.1189390

[B45] Huang S. Yin H. Lv L. (2019). Job characteristics and teacher well-being: the mediation of teacher self-monitoring and teacher self-efficacy. Educ. Psychol. 39, 313–331. 10.1080/01443410.2018.1543855

[B46] Jacobsson C. Åkerlund M. Graci E. Cedstrand E. Archer T. (2016). Teacher team effectiveness and teachers well-being. Clin. Exp. Psychol. 2, 130. 10.4172/2471-2701.1000130

[B47] Jennings P. A. Greenberg M. T. (2009). The prosocial classroom: teacher social and emotional competence in relation to student and classroom outcomes. Rev. Educ. Res. 79, 491–525. 10.3102/0034654308325693

[B48] Jones Kingsley B. (2021). Exploring the Role of Teacher Well-being on Effective Practice: A Partial Least Squares–Structural Equation Modeling Analysis. (Doctoral dissertation), ProQuest Dissertations Publishing, Manhattanville College, Harrison, NY, United States.

[B49] Katebi A. HajiZadeh M. H. Bordbar A. Salehi A. M. (2022). The relationship between “job satisfaction” and “job performance”: a meta-analysis. Glob. J. Flex. Syst. Manag. 23, 21–42. 10.1007/s40171-021-00280-y11302235

[B50] Kennedy D. (2004). The old file-drawer problem. Science 305, 451. 10.1126/science.305.5683.451 15273363

[B51] Kern M. L. Waters L. Adler A. White M. (2014). Assessing employee wellbeing in schools using a multifaceted approach: associations with physical health, life satisfaction, and professional thriving. Psychology 5, 500–513. 10.4236/psych.2014.56060

[B52] Keyes C. L. M. (2002). The Mental Health Continuum: from languishing to flourishing in life. J. Health Soc. Behav. 43, 207–222. 10.2307/3090197 12096700

[B53] Klusmann U. Aldrup K. Roloff J. Lüdtke O. Hamre B. K. (2022). Does instructional quality mediate the link between teachers' emotional exhaustion and student outcomes? A large-scale study using teacher and student reports. J. Educ. Psychol. 114, 1442–1460. 10.1037/edu0000703

[B54] Klusmann U. Kunter M. Trautwein U. Lüdtke O. Baumert J. (2008). Teachers' occupational well-being and quality of instruction: the important role of self-regulatory patterns. J. Educ. Psychol. 100, 702–715. 10.1037/0022-0663.100.3.702

[B55] Klusmann U. Waschke U. (2018). Gesundheit und Wohlbefinden im Lehrberuf [Health and Well-being in the Teaching Profession]. Göttingen: Hogrefe.

[B56] Kwon K.-A. Jeon S. Castle S. Ford T. G. (2022). Children's behavioral challenges in Head Start classrooms: links to teacher well-being and intent to leave. Early Childh. Educ. J. 50, 1221–1232. 10.1007/s10643-021-01253-7

[B57] Lavy S. Berkovich-Ohana A. (2020). From teachers' mindfulness to students' thriving: the mindful self in school relationships (MSSR) model. Mindfulness 11, 2258–2273. 10.1007/s12671-020-01418-2

[B58] Lee Y. H. Richards K. A. R. Washburn N. (2021). Mindfulness, resilience, emotional exhaustion, and turnover intention in secondary physical education teaching. Eur. Rev. Appl. Psychol. 71, 1–10. 10.1016/j.erap.2021.100625

[B59] Maas J. Schoch S. Scholz U. Rackow P. Schüler J. Wegner M. . (2022). School principals' social support and teachers' basic need satisfaction: the mediating role of job demands and job resources. Soc. Psychol. Educ. 10.1007/s11218-022-09730-6 36570365 PMC9771846

[B60] Madigan D. J. Kim L. E. (2021). Does teacher burnout affect students? A systematic review of its association with academic achievement and student-reported outcomes. Int. J. Educ. Res. 105, 101714. 10.1016/j.ijer.2020.101714

[B61] McCallum F. (2020). “The changing nature of teachers' work and its impact on wellbeing,” in Critical Perspectives on Teaching, Learning and Leadership: Enhancing Educational Outcomes, eds M. A. White and F. McCallum (Singapore: Springer Singapore), 17–44.

[B62] McCallum F. Price D. (2010). Well teachers, well students. J. Stud. Wellbeing 4, 19–34. 10.21913/JSW.v4i1.599

[B63] McCallum F. Price D. (2016). “Teacher wellbeing,” in Nurturing Wellbeing Development in Education, eds F. McCallum and D. Price (London: Routledge), 113–132.=

[B64] McCallum F. Price D. Graham A. Morrison A. (2017). Teacher Wellbeing: A Review of the Literature. Adelaide, NSW: The University of Adelaide.

[B65] Mead N. (2022). Telling tales: sharing humorous education stories to enhance teacher wellbeing and learning. Reflect. Practice 2022, 1–14. 10.1080/14623943.2022.2158797

[B66] Moè A. Katz I. (2022). Need satisfied teachers adopt a motivating style: the mediation of teacher enthusiasm. Learn. Individ. Diff. 99, 102203. 10.1016/j.lindif.2022.102203

[B67] Moher D. Liberati A. Tetzlaff J. Altman D. G. (2009). Preferred reporting items for systematic reviews and meta-analyses: the PRISMA statement. Br. Med. J. 339, b2535. 10.1136/bmj.b2535 21603045 PMC3090117

[B68] Mottet T. P. Beebe S. A. (2000). “Emotional contagion in the classroom: an examination of how teacher and student emotions are related,” in Paper presented at the Annual Meeting of the National Communication Association. Seattle, WA.

[B69] Naghieh A. Montgomery P. Bonell C. P. Thompson M. Aber J. L. (2015). Organisational interventions for improving wellbeing and reducing work-related stress in teachers. Cochr. Database Systemat. Rev. 4, CD010306. 10.1002/14651858.CD010306.pub2 25851427 PMC10993096

[B70] Olsen S. T. Andreassen Becher A. Bergflødt S. Hammer A. Paaske N. Palm K. . (2021). “Teacher well-being and teacher professional development,” in International Perspectives on Teacher Well-being and Diversity: Portals Into Innovative Classroom Practice, eds T. R. N. Murphy and P. Mannix-McNamara (Singapore: Springer Nature), 87–118. 10.1007/978-981-16-1699-0_535967696

[B71] Ostroff C. (1992). The relationship between satisfaction, attitudes, and performance: an organizational-level analysis. J. Appl. Psychol. 77, 963–974. 10.1037/0021-9010.77.6.96319186900

[B72] Page M. J. McKenzie J. E. Bossuyt P. M. Boutron I. Hoffmann T. C. Mulrow C. D. . (2021). The PRISMA 2020 statement: an updated guideline for reporting systematic reviews. Br. Med. J. 372, n71. 10.1136/bmj.n71 33782057 PMC8005924

[B73] Pap Z. Maricu?oiu L. Vîrgă D. Ilie M. Mladenovici V. Popescu B. . (2023). Happy teacher, healthy class? Linking teachers' subjective well-being to high-school and university students' physical and mental health in a three-level longitudinal study. Soc. Psychol. Educ. 23, 9768. 10.1007/s11218-023-09768-0

[B74] Pfleging A. C. (2022). Confronting the Challenges of the COVID-19 Pandemic: A Quantitative Analysis of the Relationships Among Secondary Teachers' Perceived Resilience, Trait Emotional Intelligence, and Self-efficacy. (Doctoral dissertation), ProQuest Dissertations Publishing, Manhattanville College, Harrison, NY, United States.

[B75] Pugliese C. (2019). A Study of the Influence of Bidirectional Teacher–Student Relationships on Teacher Wellbeing. (Doctoral dissertation), ProQuest Dissertations Publishing, Neumann University, Aston, PA, United States.36421629

[B76] Roffey S. (2012). Pupil wellbeing – teacher wellbeing: two sides of the same coin? Educ. Child Psychol. 29, 8–17. 10.53841/bpsecp.2012.29.4.8

[B77] Ryan R. M. Deci E. L. (2016). “Facilitating and hindering motivation, learning, and well-being in schools: research and observations from self-determination theory.” in Handbook of Motivation at School, eds K. R. Wentzel and D. B. Miele (New York, NY: Routledge), 96–119.

[B78] Ryff C. D. (1989). Happiness is everything, or is it? Explorations on the meaning of psychological well-being. J. Personal. Soc. Psychol. 57, 1069–1081. 10.1037/0022-3514.57.6.1069

[B79] Sánchez Solarte A. (2022). Teachers Matter: Exploring Foreign Language Teachers' Well-being, Their Instructional Practices, and Their Links to Student Engagement. (Doctoral dissertation), ProQuest Dissertations Publishing, Florida State University, Tallahassee, FL, United States.

[B80] Sandilos L. E. Neugebauer S. R. DiPerna J. C. Hart S. C. Lei P. (2022). Social-emotional learning for whom? Implications of a universal SEL program and teacher well-being for teachers' interactions with students. School Mental Health 22, 9543. 10.1007/s12310-022-09543-0 36188165 PMC9510330

[B81] Seligman M. E. P. (2011). Flourish: A Visionary New Understanding of Happiness and Well-being. Free Press, Detroit, Michigan, United States.

[B82] Shoshani A. (2021). Growth mindset in the maths classroom: a key to teachers' well-being and effectiveness. Teach. Teaching 2021. 2007370. 10.1080/13540602.2021.2007370

[B83] Silvia S. Panari C. Guglielmi D. Fraccaroli F. (2012). Teachers' well-being and effectiveness: the role of the interplay between job demands and job resources. Procedia 79, 729–738. 10.1016/j.sbspro.2012.11.467

[B84] Skaalvik E. M. Skaalvik S. (2018). Job demands and job resources as predictors of teacher motivation and well-being. Soc. Psychol. Educ. 21, 1251–1275. 10.1007/s11218-018-9464-833143180

[B85] Tikkanen L. Pyhältö K. Soini T. Pietarinen J. (2021). Crossover of burnout in the classroom—Is teacher exhaustion transmitted to students? Int. J. School Educ. Psychol. 9, 326–339. 10.1080/21683603.2021.1942343

[B86] Turner K. Thielking M. (2019). Teacher wellbeing: its effects on teaching practice and student learning. Iss. Educ. Res. 29, 938–960.

[B87] Turner K. Thielking M. Meyer D. (2021). Teacher wellbeing, teaching practice and student learning. Iss. Educ. Res. 34, 1293–1311.

[B88] Van Petegem K. Aelterman A. Rosseel Y. Creemers B. (2007). Student perception as moderator for student wellbeing. Soc. Indicat. Res. 83, 447–463. 10.1007/s11205-006-9055-5

[B89] Viac C. Fraser P. (2020). Teachers Well-being: A Framework for Data Collection and Analysis. OECD Education Working Paper No. 213. Organisation for Economic Co-operation and Development. Available online at: http://www.oecd.org/officialdocuments/publicdisplaydocumentpdf/?cote=EDU/WKP(2020)1&docLanguage=En (accessed February 18, 2023).

[B90] Virtanen T. E. Vaaland G. S. Ertesvåg S. K. (2019). Associations between observed patterns of classroom interactions and teacher wellbeing in lower secondary school. Teach. Teacher Educ. 77, 240–252. 10.1016/j.tate.2018.10.013

[B91] Vo D. T. Allen K.-A. (2022). A systematic review of school-based positive psychology interventions to foster teacher wellbeing. Teach. Teaching 28, 964–999. 10.1080/13540602.2022.2137138

[B92] Walter H. L. (2020). Exploring Early Childhood Special Education Teachers' Wellbeing Through a Multidimensional Framework: A Mixed-Methods Study. (Doctoral dissertation), ProQuest Dissertations Publishing, George Washington University, Washington, DC, United States.

[B93] Wang H. Hall N. C. (2021). Exploring relations between teacher emotions, coping strategies, and intentions to quit: a longitudinal analysis. J. School Psychol. 86, 64–77. 10.1016/j.jsp.2021.03.005 34051918

[B94] Wang H. Hall N. C. King R. B. (2021). A longitudinal investigation of teachers' emotional labor, well-being, and perceived student engagement. Educ. Psychol. 2021, 1988060. 10.1080/01443410.2021.198806027409075

[B95] Warr P. (1999). “Well-being and the workplace,” in Well-Being: The Foundations of Hedonic Psychology, eds D. Kahneman, E. Diener, and N. Schwarz (New York: Russel Sage), 392–412.

[B96] Yousefi-Nooraie R. Shakiba B. Mortaz-Hejri S. (2006). Country development and manuscript selection bias: a review of published studies. BMC Med. Res. Methodol. 6, 37. 10.1186/1471-2288-6-37 16879753 PMC1550721

[B97] Zewude G. T. Hercz M. (2022). The role of positive psychological capital in the prediction of teachers' well-being mediated through motivation: a review of literature. Athens J. Health Med. Sci. 9, 245–264. 10.30958/ajhms.9-4-4

[B98] Zheng S. Liu H. Yao M. (2022). Linking young teachers' self-efficacy and responsibility with their well-being: the mediating role of teaching emotions. Curr. Psychol. 22, 3342. 10.1007/s12144-022-03342-1

